# Why followership matters in psychiatry: rebalancing our obsession with leadership

**DOI:** 10.1192/bjo.2026.11037

**Published:** 2026-04-24

**Authors:** Richard A. Laugharne, Regi T. Alexander, Kenneth R. Kaufman, Derek K. Tracy, Fabida Aria, Mayura Deshpande, Matthew Frost, Rohit Shankar

**Affiliations:** Cornwall Intellectual Disability Equitable Research (CIDER), Peninsula Medical School, University of Plymouth, Truro, UK; Adult Intellectual Disability Service, Cornwall Partnership NHS Foundation Trust, Truro, UK; Adult Intellectual Disability Service, Hertfordshire Partnership NHS Foundation Trust, Hatfield, UK; Department of Psychiatry, Rutgers Robert Wood Johnson Medical School, New Jersey, US; Department of Psychiatry, South London and Maudsley NHS Foundation Trust, London, UK; Department of Psychosis Studies, Institute of Psychiatry Psychology and Neuroscience, London, UK; Department of Psychiatry, Birmingham and Solihull Mental Health NHS Foundation Trust, Birmingham, UK; Department of Psychiatry, Hampshire and Isle of Wight Healthcare NHS Foundation Trust, Southampton, UK; Plymouth Marine Laboratory, University of Plymouth, Plymouth, UK

**Keywords:** Education and training, ethics, mental health services, patients and service users, philosophy

## Abstract

Psychiatry emphasises leadership development, but neglects the equally universal and essential role of followership. Although most clinicians spend more time as followers than leaders, literature overwhelmingly favours leadership. Drawing on healthcare, socio-religious traditions and management science, the authors reframe followership as an active, values-driven role grounded in trust, motivation and moral courage rather than passive compliance. This editorial argues that effective followership is active, ethical and courageous. Nurturing good followers in mental health services is essential for good patient care, organisational integrity and sustainable leadership. Cultivating active followership strengthens safeguarding, transparency and organisational legitimacy. The editorial calls for psychiatry and mental health services to explicitly teach and value followership alongside leadership, promoting shared vision, psychological safety and accountable decision-making to improve patient care and professional culture.

**Figure:**
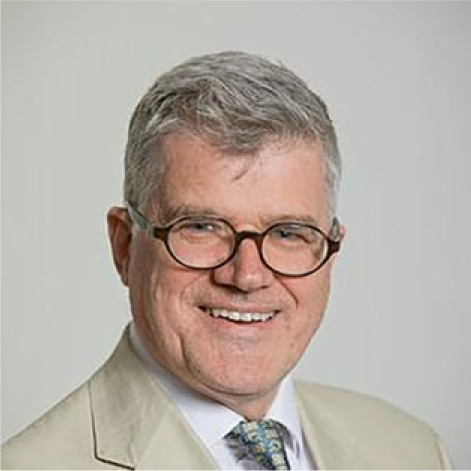

As psychiatrists, we are continually being encouraged to become good leaders. There are books and courses on how to develop leadership skills, and indeed, there has been a helpful and needed growth in the ‘professionalisation’ of clinical leadership, such as the formation of the pan-Royal College Faculty of Medical Leadership and Management with training and accreditation standards, and various Fellowship schemes. However, one thing that we have all been and will continue to be is a follower. Very few of us are not accountable to someone above us in a hierarchy.

A leader can only lead if others are willing to follow. We can only be a follower if someone else is willing to lead. The experience of being a follower in work settings begins on our first day of employment and is universal, but many of us working in health, research and science will be both followers and leaders, often at the same time.

A 2004 review revealed 95 220 titles devoted to leadership and 792 titles on followership, with most of the latter being in political and spiritual spheres.^1^ Another review in healthcare found 60 times more papers on leadership than on followership.^2^ To some, being a follower can be stigmatising. Followers can be characterised negatively as passive, weak and conforming, and choose not to define themselves as such.^3^ Surely developing good followers in healthcare is as important, if not more important, than trying to develop leaders?

## Followership: a socio-religious perspective

It has been a recognised, respected position of education and erudition throughout history to learn from and follow those with perceived wisdom. Canonically, of the three foundational Greek philosophers, Aristotle was the student and follower of Plato, who had been a follower of Socrates.

In theology, followership is a frequent topic of debate and can have a much more positive image included in the term discipleship. Discipleship has been considered a similar principle in Hinduism, Judaism, Islam and Christianity.^4^ In Christianity, the origins of a disciple following a rabbi come from first century Judaism, and the term disciple can be interpreted as follower, pupil or apprentice. The art of discipleship was an active following of the rabbi (teacher), submission to the leadership of another. Leadership is reconsidered as an act of service, becoming a servant to others, and the word minister, which in the UK is used as a term for political leaders as well as religious leaders, has its etymological root in the Latin word for servant.

There may be a more positive view of followership in a socio-religious context, but this has a darker side. The abuse of power by religious leaders is commonly reported in the press, which, at its most extreme, can result in controlling behaviour, sexual abuse, violence and mass suicide. The assent of followers can be mystifying, but the pattern of followers ignoring or colluding with bad actions at the request of charismatic religious leaders is too common to be disregarded. The establishment of safeguarding principles in religious, educational, occupational, voluntary, sporting and other spheres suggest that these problems in the leadership–followership dynamic are universal. Healthcare, scientific and research settings are far from immune to this phenomenon, and therefore need robust safeguarding measures.

## Management science and followership

Managerial literature on followership identifies three theoretical areas:

- Follower motivations: The follower’s motivation includes internal and environmental factors. Motivation is generated internally through reflection on how hard they will work, what recognition or reward they will get and whether that reward will be worth it.^5^ They need to have confidence that they can do the job expected of them, trust the leaders to tie outcome to performance, and gain satisfaction with the outcomes they receive.
- Follower values and trust: There needs to be a congruence of values and mutual trust between follower and leadership. This distinguishes following from complying, the former is driven by values and the latter by rules-based compliance.
- Effective and ineffective followers: Research suggests effective followers manage themselves well, are committed to a purpose beyond themselves, build their competence to have maximum impact and strive to be courageous, honest and credible.^6^ A willingness to tell the truth is crucial.

Five types of followers are described:^6^

- Effective: A follower who is both an independent thinker and active in behaviour. They exhibit consistent behaviour with all people, regardless of their position in the organisation, and deal well with conflict and risk. They are comfortable with change, put forward their own views, and stay focused on the needs of the organisation. They understand how others see them. They are able to make acts of leadership and use their referent (the ability of a leader to influence a follower), expert, network and information power in service of the organisation. This group are ‘effective followers’, and they can be viewed as ‘leaders in waiting’.
- Conformist: This follower type is busy, but does not necessarily think through what it is they are doing. They participate willingly, but do not question orders. They will avoid conflict at all costs and take the quietest path, but will loyally defend their boss to extremes. This follower type is ‘the yes-people’.
- Passive: This follower type can be the result of excessive micro-management or a negative, over-controlling and blame-oriented culture. They do not show any initiative nor take responsibility. This is the passive follower. This group is ‘the sheep’.
- Alienated: This follower thinks well, but for some reason, often snipes from the sidelines. They have got stuck where they are, can be very negative and feel they have lost their power. They have become bitter in their work from being passed over for promotion, or from having stayed too long in one position. They are ‘the alienated followers’.
- Pragmatic survivor: This follower type can be seen as the organisational ‘canary in the coal mine’. They can flip between different followership styles to suit each situation, and are our early warning system when the culture of the organisation is starting to change for the worse. They are ‘survivors’.

## Followership in healthcare

A systematic review on leadership and followership in health professions included 81 articles, with 46 concentrating on followership qualities.^7^ Forty-eight articles were theoretical and only 22 were empirical. They found similar, but not identical, qualities in being a good leader and a good follower (Table 1). Only one article in the review was in mental healthcare, and that was in a military context and not from a health administrative perspective.^8^

**Table 1:** Number of articles discussing qualities of leadership and followership^7^

Qualities	Leadership	Followership
Effective communication	16	8
Visionary	14	0
Delegating tasks	9	0
Situational awareness	7	0
Providing/receiving feedback	7	7
Supportive	7	10
Flexibility	5	2
Trustworthy	5	5
Adaptability	5	0
Empathetic	5	0
Emotional intelligence	4	0
Critical thinking	3	9
Proactive	3	3
Responsible	0	16
Committed	0	11
Take the initiative	0	5

A state-of-the-art narrative review of followership in inter-professional healthcare teams identified two contradictory views of followership.^9^ The first, as active team members with shared leadership emphasised and the second as passive team members. This review suggested that for an emphasis on distributed, shared and situational leadership models, there needs to be a harnessing of a renewed understanding of active followership.

A scoping review mapping leadership and followership in primary care for patients with chronic illness found a shifting picture of roles between physicians and other health professionals.^10^ The findings again emphasised that followership should be an active role and not passive, and all members of staff in integrated healthcare teams should seek to be able to adopt both roles according to different situational demands.

## Followership in psychiatry and mental healthcare

A focus on followership may be beneficial for patient care, organisational functioning and career satisfaction, but suffers from a dearth of research.^7,8^ Educating staff on the importance of being a good follower may be beneficial, emphasising that followership is not evidence of a lack of ambition, but an active requirement of everyone.

Psychiatrists (trainees and consultants) and other clinicians switch between leadership and followership roles as a matter of course, both within and between clinical and non-clinical positions (such as in research, management and education). There is an opportunity to use leadership/followership models training to adapt positively between roles, raising morale and preventing burnout. We suggest that leadership courses should develop into leadership/followership courses.

For psychiatrists, the symbiotic relationship between leaders and followers needs to be actively nurtured. Institutional bodies such as the Royal College of Psychiatrists democratically elect their leaders. With members being dispersed across differing communities, geography and levels of resource, a dislocation between members and central leadership can occur. The active nurturing of networks and connection is a challenging but necessary task, and needs to be constantly reviewed. Decision making needs to be explained, transparent and accountable. Our leaders are empowered to make many decisions on our behalf, but a spirit of transparency and accountability is vital to foster trust.

Psychiatry is practised within complex hierarchies in which most clinicians are, simultaneously and repeatedly, both leaders and followers. Yet while leadership is celebrated and taught, followership remains poorly articulated and undervalued. Effective followership in psychiatry is not passive compliance, but an active, ethical and courageous stance, requiring critical thinking, responsibility and a willingness to speak uncomfortable truths in the service of patients and professional values. Poor followership risks disengagement, moral injury and the silent normalisation of unsafe practice, whereas good followership strengthens trust, safeguarding and organisational legitimacy.^11,12^ Psychiatry would benefit from explicitly recognising, teaching and valuing active followership alongside leadership, not as a lesser role, but as a marker of professional maturity and a prerequisite for humane, accountable and effective mental healthcare.

In healthcare, we have many layers of leaders and organisations with unwritten rules that the followers need to follow as told. Many feel disenfranchised to speak up or share ideas as they feel they are not in a leadership position. The best leaders will elicit these ideas and ensure they are shared while acknowledging that the best qualities of followers are helping to make the changes needed.

Having a common vision to improve patient care and experience for leaders and followers can truly revolutionise healthcare. This is possible in teams that have the culture to speak openly and where there is psychological safety, and the ability to adapt, change and continuously improve.

With a public healthcare system where productivity and not quality of care is being primarily monitored, coming together and speaking on the need for more focus on quality and patient experience is a requirement for leaders and followers. The best followers are those who also have their own unique leadership qualities, where they can challenge a leader, and the leader in turn utilises these challenges in a way that helps everyone improve. The collective power of followers is not to be underestimated, and staying true to their values and vision can make all the difference.

In conclusion, we are all followers, but we are most effective at following when we are led by those whose values chime with ours. Being inspired is a pleasurable experience, and effective followers help to create meaningful leaders. The gentle tug of active followers who refuse to abandon what is true will always be more authentic than the bluster of leaders who rediscover truth only when convenient.
